# Comparative analysis of gut viromes in four penguin species reveals diverse novel viruses and host-associated differences

**DOI:** 10.1128/msphere.00848-25

**Published:** 2026-06-30

**Authors:** Kang Qi, Shuang Zhang, Xiaodong Su, Jiaheng Chen, Shiyin Huang, Yue Chen, Wang Li, Guanying Ni, Jieji Duo, Shixing Yang, Quan Shen, Xiaochun Wang, Yuwei Liu, Ping Wu, Hongfeng Yang, Likai Ji, Xiaolong Wang, Wen Zhang

**Affiliations:** 1 Institute of Critical Care Medicine, The Affiliated People’s Hospital, Jiangsu University 12676, Zhenjiang, China; 2 Department of Laboratory Medicine, School of Medicine, Jiangsu University 191611, Zhenjiang, China; 3 Key Laboratory of Wildlife Diseases and Biosecurity Management of Heilongjiang Province, Harbin, Heilongjiang, China; 4 Sino-Ethiopian Wildlife Disease Research Joint Laboratory, Harbin, Heilongjiang, China; 5 Tianjun County Center for Disease Control and Prevention, Tianjun, Qinghai, China; 6 The Haixi Extension Service Center for Agricultural and Animal Husbandry Technology, Delingha, Qinghai, China; 7 The Haixi Forest Pest Control and Quarantine Center, Delingha, Qinghai, China; University of Wisconsin-Madison, Madison, Wisconsin, USA

**Keywords:** viral metagenomics, penguins, gut viromes, bacteriophages

## Abstract

Penguins, as distinctive marine birds, play important roles in polar and sub-Antarctic ecosystems, yet the diversity and species-specific distribution of their gut viromes remain insufficiently understood. Here, we used viral metagenomics to characterize the cloacal viromes of four penguin species—*Spheniscus humboldti* (*S. humboldti*), *Pygoscelis papua* (*P. papua*), *Pygoscelis adeliae* (*P. adeliae*), and *Aptenodytes forsteri* (*A. forsteri*)—collected at Chimelong Ocean Kingdom. A total of 219 viral sequences representing potentially novel lineages were identified, with more than 94% sharing <80% amino acid similarity with previously known viruses. These sequences were assigned to several viral families, including Parvoviridae, Caliciviridae, Anelloviridae, Circoviridae, and Microviridae, among others. Marked interspecies differences in virome composition were observed: Parvoviridae dominated in *S. humboldti*, Microviridae were enriched in *P. papua*, Caliciviridae accounted for a substantial proportion in *A. forsteri*, and *P. adeliae* displayed the greatest overall virome diversity. Multiple-virus co-detections, particularly involving Parvoviridae, were frequent in *S. humboldti*. Phylogenetic analyses showed that many penguin-associated viruses clustered with viruses infecting other avian and fish hosts, suggesting possible dietary or environmental origins of some detected viral sequences. These findings expand current knowledge of penguin gut virome diversity and host-associated differences and provide a valuable foundation for evaluating the ecological roles, health implications, and transmission risks of penguin-associated viruses.

**IMPORTANCE:**

This study uncovers significant diversity in the gut viromes of four penguin species, revealing over 219 viral sequences representing potentially novel lineages, many of which showed host-associated distribution patterns. Using viral metagenomics, we identified notable interspecies differences, with Parvoviridae predominating in Spheniscus humboldti and Microviridae being enriched in Pygoscelis papua. These findings highlight the complexity of viral community structures in penguins, including frequent viral co-detections, which could impact host health and ecological adaptation. Additionally, novel bacteriophage communities were identified, emphasizing their potential role in shaping the gut microbiome and influencing viral dynamics. This work provides new insights into viral diversity in wildlife and lays the groundwork for future studies on viral transmission risks and ecological conservation.

## INTRODUCTION

Penguins (Order: Sphenisciformes) are marine birds found across temperate regions of the Southern Hemisphere, from the Antarctic continent to the Galápagos Islands (1). As important components of the food chain, penguins play a vital role in marine ecosystems and may serve as hosts for various pathogens (2). However, the diversity of the penguin gut virome and its interspecies variation are underexplored, limiting our understanding of viruses’ roles in penguin health, ecological adaptation, and potential cross-species transmission (3, 4).

Microbial infections have contributed to declines in bird populations worldwide, including the reduction of North American passerines due to West Nile virus, Hawaiian birds from avian malaria, and albatrosses from avian cholera (5–7). Compared to mammals and other birds, the composition and diversity of penguin gut viromes remain poorly understood. Previous studies have primarily focused on symptomatic individuals in natural settings (8), with traditional diagnostic methods, such as serology and PCR, restricting detection to known viruses. Viral metagenomics, however, has overcome these limitations, revealing numerous novel viruses. Evidence suggests that penguins may harbor both common avian viruses and new, previously unidentified viral species. Since 2016, over 20 novel viruses have been identified in Antarctic Penguins (9), spanning families such as Astroviridae, Adenoviridae, Circoviridae, Caliciviridae, Herpesviridae, Coronaviridae, Papillomaviridae, Orthomyxoviridae, Paramyxoviridae, Polyomaviridae, Reoviridae, and Picornaviridae (2, 10–13). Similar viral families have been detected in penguins outside Antarctica, including *African*, *Magellanic*, and *little blue* penguins, indicating a broader viral diversity that warrants further investigation (14, 15). Additionally, co-detections are common in penguin gut viromes, which may increase disease burden and promote viral recombination, potentially leading to new viral strains (16). Currently, research on gut viral communities and viral co-detections in penguins is limited, making systematic investigations essential for understanding their pathogen profiles and co-detection dynamics.

Bacteriophages also play a significant role in the gut virome (17). Although they do not directly infect hosts, bacteriophages influence microbial community structure, regulate bacterial populations, and may facilitate the horizontal transfer of antibiotic resistance genes (18). These indirect effects can affect host health and increase vulnerability to opportunistic pathogens (19). Recent metagenomic studies have uncovered vast amounts of “viral dark matter” (20), suggesting that bacteriophages, even in the absence of identified pathogens, can contribute to microbial dysbiosis or facilitate gene transfer (21). Thus, studying bacteriophages in penguin viromes is equally important.

This study aims to characterize the gut viromes of *Spheniscus humboldti*, *Pygoscelis papua*, *Pygoscelis adeliae*, and *Aptenodytes forsteri*. Cloacal samples from five individuals per species (a total of 20 samples) were collected at the Chimelong Ocean Kingdom. Using viral metagenomics and high-throughput sequencing, we analyzed the composition and phylogenetic features of their gut viromes, focusing on Parvoviridae, Caliciviridae, Circoviridae, Anelloviridae, Picornaviridae, and bacteriophage communities. By comparing viral diversity and co-detection patterns, we aim to reveal species-specific viral community differences and assess their ecological and health implications. This study provides critical data for understanding penguin viral diversity, supporting viral surveillance, ecological conservation, and pathogen transmission risk assessment.

## RESULTS

### Overview of the virome

In this study, we collected 20 cloacal samples from four penguin species (*Spheniscus humboldti*, *Pygoscelis papua*, *Pygoscelis adeliae*, and *Aptenodytes forsteri*) at the same zoo for viral metagenomic analysis, examining both DNA and RNA viruses separately. Each sample underwent independent viral particle enrichment, nucleic acid extraction, and library construction, resulting in the preparation of 20 libraries. Following next-generation sequencing on the Illumina NovaSeq platform, reads associated with archaea, bacteria, eukaryotes, and other non-viral particles were removed, yielding 192,621,860 raw reads (averaging 9,631,093 per library) with an average read length of 151 bp and a GC content of 43.09% (Table S3).

### Characterization of viral communities

To elucidate the composition and distribution of the gut virome in the four penguin species, viral metagenomic analysis was performed on 20 cloacal samples (five from each species). The species rarefaction curves showed that as sequencing reads increased, the number of viral species detected in most libraries gradually approached a plateau, indicating that the current sequencing depth was sufficient to capture the majority of viral diversity present in the samples (Fig. S1). Similarly, the species accumulation curves exhibited a saturation trend as additional samples were included, suggesting that the current sampling size was adequate to represent most viral species richness in the data set (Fig. S2). Together, these results indicate that further increases in sequencing depth or sample number would be unlikely to substantially increase the number of detected viral species. In total, over 350 viral species were detected across all libraries, reflecting high species diversity in the penguin gut virome.

**Fig 1:**
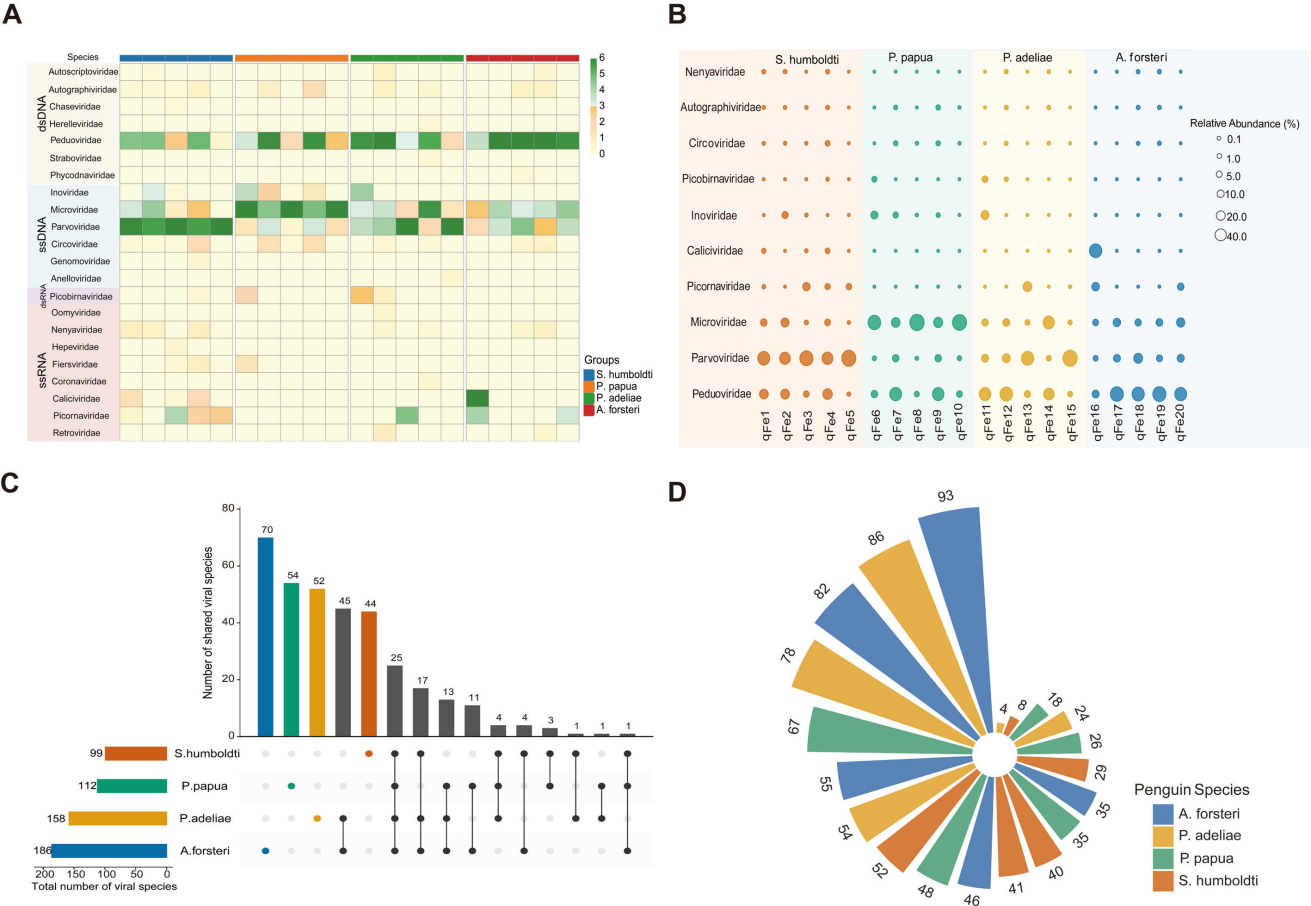
Taxonomic analyses of viral reads at the family or species level. (**A**) Heatmap representing the read counts of each viral family in each pool on a log10 scale. Viral types, viral families, and groups are annotated with corresponding colors (see color legend). (**B**) A bubble chart showing the top 10 most abundant viral families. Bubble size indicates the abundance of reads assigned to each family. (**C**) UpSet plot depicting the numbers of shared viral species among the four viromes. Filled spots with interconnecting vertical lines represent sharing between the corresponding libraries. (**D**) A Nightingale rose diagram illustrating the number of species in each of the 20 pools (see color legend).

At the family level, the heatmap revealed that viruses were primarily classified into 22 viral families, including 13 DNA viral families (7 double-stranded DNA families and 6 single-stranded DNA families) and 9 RNA viral families (Fig. 1A). Among them, Parvoviridae, Microviridae, and Peduoviridae were among the most abundant viral families. Parvoviridae was particularly predominant in *Spheniscus humboldti*, while Caliciviridae showed specific enrichment in sample qFe16, and Picornaviridae was more prominent in samples qFe3, qFe13, and qFe16. Bubble plot analysis indicated that Peduoviridae was evenly distributed across the four species, Parvoviridae was most abundant in *S. humboldti*, Microviridae dominated in *P. papua*, and Caliciviridae was also present in *A. forsteri* (Fig. 1B). At the species level, 555 viral species were identified, with 99, 112, 158, and 186 viral species detected in *S. humboldti*, *P. papua*, *P. adeliae*, and *A. forsteri*, respectively. Among these, each species harbored 44, 54, 52, and 70 unique species, respectively (Fig. 1C). Notably, only 25 viral species were shared among the four species, accounting for 13%–25% of the total species per host, indicating significant host-associated differences in the penguin gut virome. A Nightingale rose diagram revealed the highest species abundance in qFe18 and the lowest in qFe15. Overall, *A. forsteri* samples exhibited the highest species abundance, while *S. humboldti* samples showed the lowest (Fig. 1D; Table S4).

**Fig 2:**
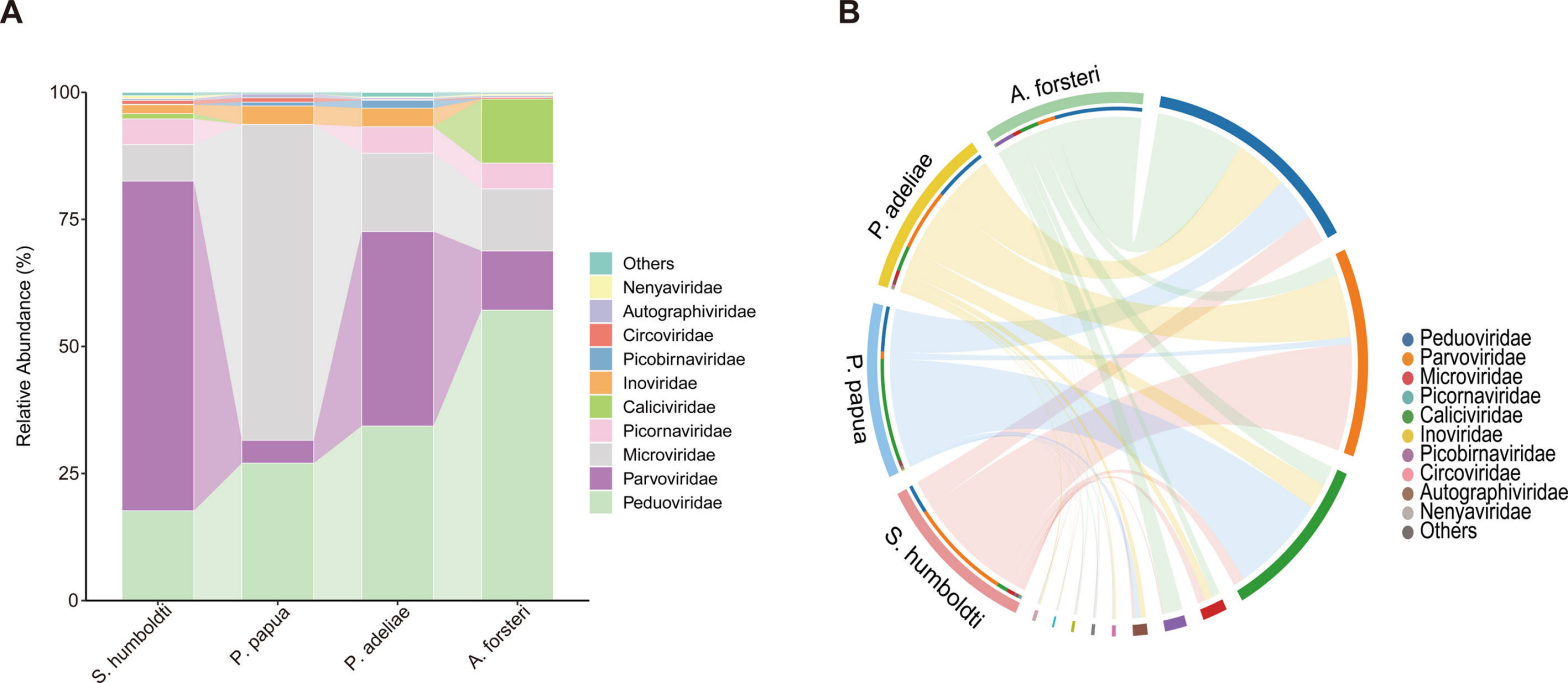
Composition of viral species in penguin cloacal samples. (**A**) Stacked bar chart showing the relative abundance of the top 10 viral families in the samples. (**B**) Chord diagram illustrating the relative abundance of the top 10 viral families in the eukaryotic viromes of the four penguin species. The length of each arc represents the proportion of each viral family, with different colors indicating different families.

To compare the compositional differences of gut viromes among the four species, we generated a relative abundance stacked bar plot (Fig. 2A). The results revealed that Parvoviridae was the most abundant viral family in *S. humboldti*, while Microviridae exhibited a significant abundance in *P. papua*. Additionally, Peduoviridae, belonging to the class Caudoviricetes, was present in all four species, with its highest proportion in *A. forsteri*. In contrast, the gut virome composition of *P. adeliae* was more diversified, with no single dominant viral family. A chord diagram (Fig. 2B) further elucidated the shared and specific distribution of viral communities among the penguin hosts. Peduoviridae, Parvoviridae, and Microviridae were shared among all species, suggesting that these families may constitute the core gut virome in penguins. Certain viral taxa, such as Microviridae, were more closely associated with *P. papua*, while Parvoviridae was particularly linked with *S. humboldti*. Low-abundance viral families, such as Caliciviridae and Inoviridae, exhibited host-associated distribution patterns, further highlighting structural differences in gut viral communities.

**Fig 3:**
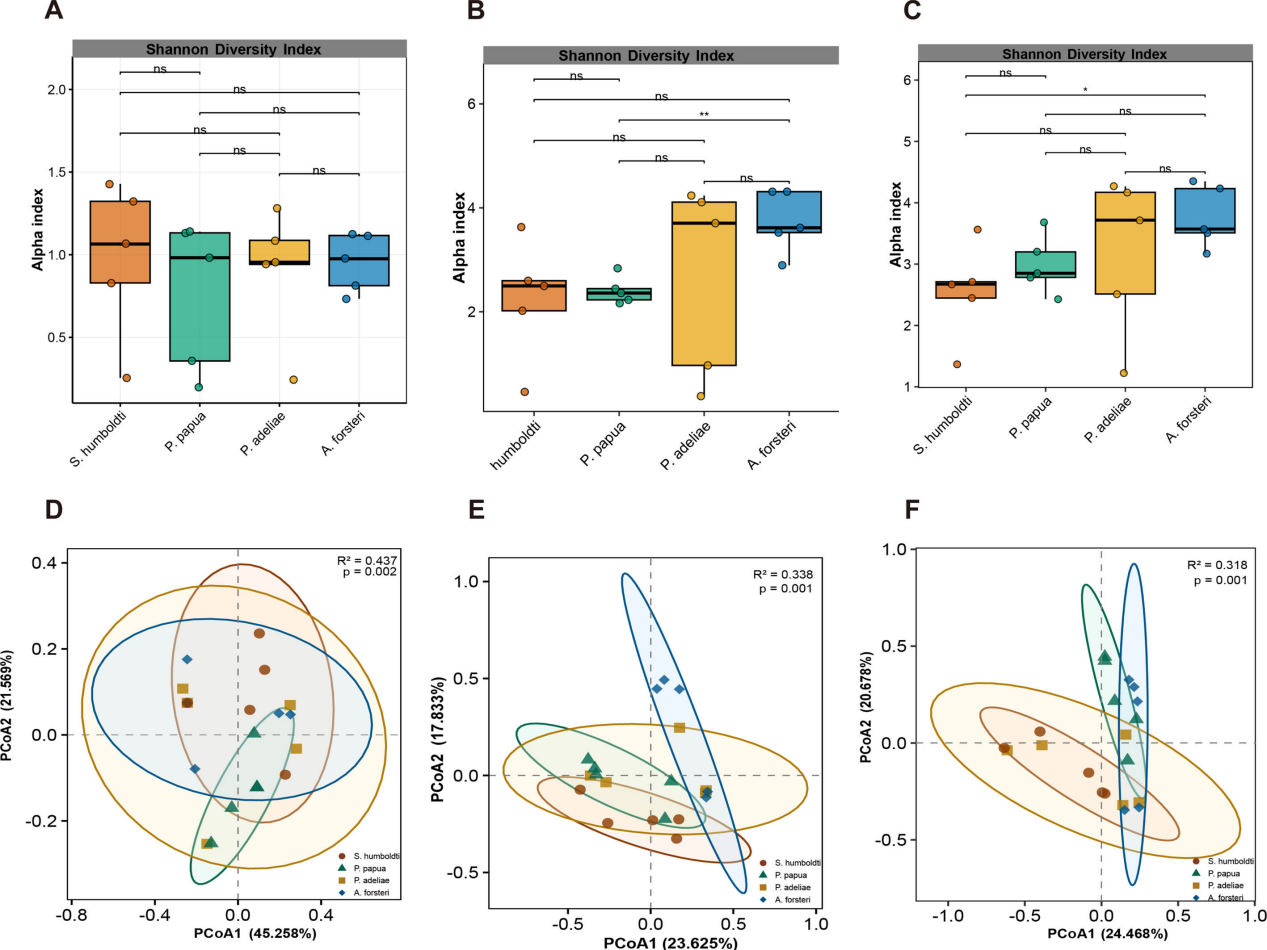
Diversity of the penguin gut viral communities. (**A–C**) Comparison of viral α-diversity, normalized by MEGAN, measured using the Shannon index based on viral abundance at the family level. The *P*-value was calculated using the Wilcoxon test. Horizontal bars represent medians, with the top and bottom of the boxes indicating the 75th and 25th percentiles, respectively. ns, no significant difference, *P* < 0.05 (*Spheniscus humboldti* vs *Aptenodytes forsteri*), *P* < 0.01 (*Pygoscelis papua* vs *Aptenodytes forsteri*). (**D–F**) Comparison of viral β-diversity, normalized by MEGAN, with PCoA of viral communities at the family level. A *P*-value of <0.05 was considered statistically significant.

Diversity comparisons using the Shannon index revealed no significant differences in viral alpha diversity among penguin species at the family level (*P* > 0.05) (Fig. 3A), whereas significant differences were observed at finer taxonomic resolutions. Specifically, the Shannon index differed significantly between *P. papua* and *A. forsteri* at the genus level (Fig. 3B) and between *S. humboldti* and *A. forsteri* at the species level (Fig. 3C). To further examine differences in community composition, beta diversity was assessed using principal coordinates analysis (PCoA) based on unweighted UniFrac distances (Fig. 3D through F). The ordination plots showed separation among viral communities from different penguin species, with the *P. adeliae* samples displaying the greatest dispersion and the *P. papua* samples showing relatively limited variation. PERMANOVA analysis further confirmed that gut viral community composition differed significantly among penguin species at the family, genus, and species levels (*P* = 0.002, 0.001, and 0.001, respectively) (Fig. 3D through F), indicating host-associated differences in viral community structure among penguin species.

### Identification of viral sequences

A total of 219 viral sequences were obtained after contig extension and annotation. These sequences were primarily assigned to seven highly abundant viral groups, each encoding signature genes for their respective taxa. These included the major capsid protein (MCP) for Microviridae, non-structural protein 1 (NS1) for Parvoviridae, replication-associated protein (Rep) for Circoviridae, RNA-dependent RNA polymerase (RdRp) for Picornaviridae, capsid protein (CP) for Caliciviridae, capsid protein VP1 for Anelloviridae, and large terminase subunit (TERL) for Caudoviricetes. BLASTx analysis showed that the similarity between these sequences and their best-matching homologs ranged from 34.6% to 99.3%. The classification of viral sequences followed the current ICTV (International Committee on Taxonomy of Viruses) taxonomy framework for each viral family and was evaluated primarily based on the amino acid similarity of key proteins together with phylogenetic relationships, rather than a single universal similarity threshold.

### Phylogenetic analyses of main virus groups

#### Phylogeny and host distribution of Parvoviridae

Parvoviridae is a group of non-enveloped, single-stranded DNA viruses with a genome length of approximately 4–6 kb, containing two major open reading frames (ORFs) (22). This family is divided into three subfamilies: Densovirinae (infecting invertebrates), Parvovirinae (infecting vertebrates), and Hamaparvovirinae (infecting both vertebrates and invertebrates) (23). Although Parvoviridae have been reported in various bird species(24), research on their distribution in seabirds, especially penguins, is limited.

**Fig 4:**
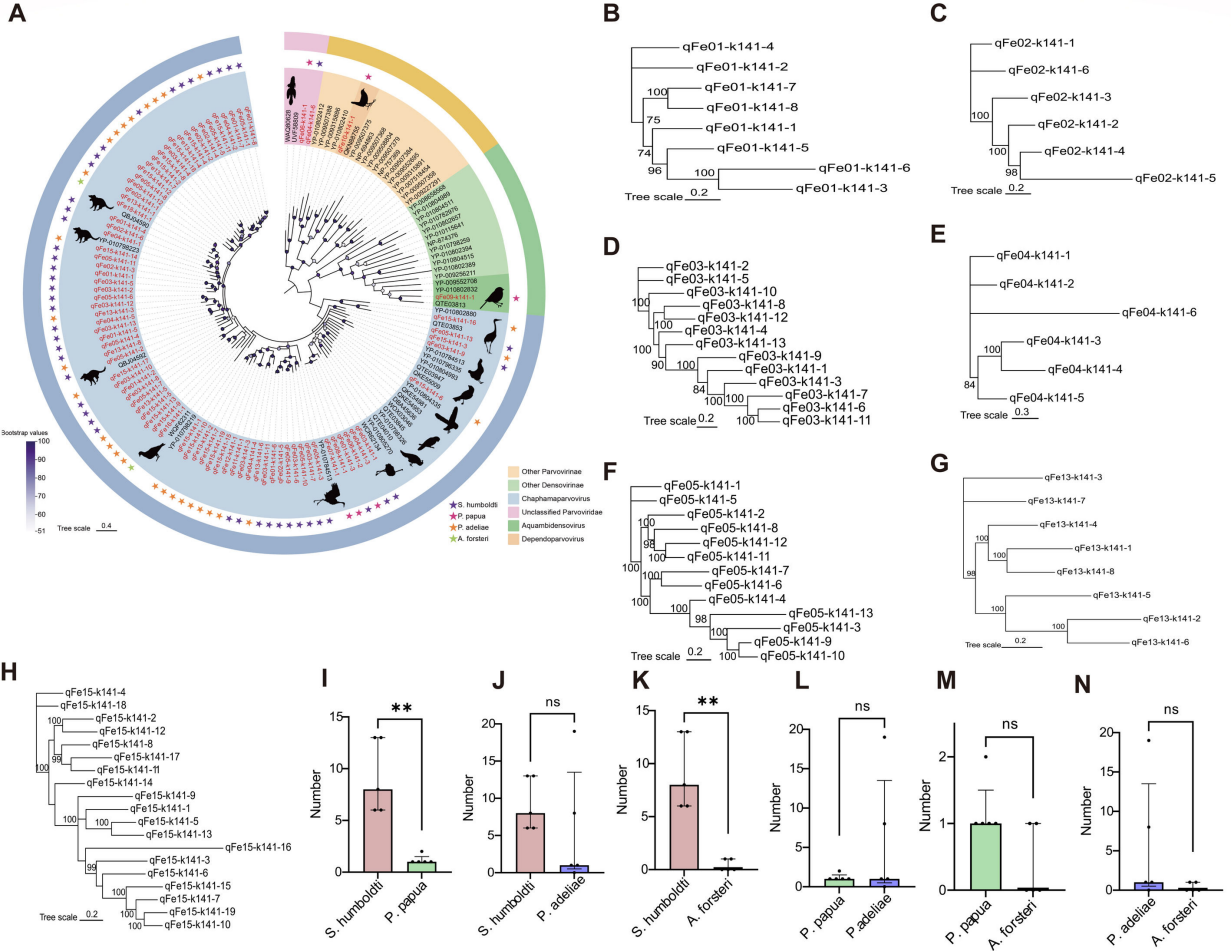
Phylogenies of parvoviruses identified in the cloaca of penguins. (**A**) Maximum likelihood tree based on NS1 protein amino acid sequences. Sequences obtained in this study are highlighted in red. Support values are shown on the branches, with the scale bar representing substitutions per site. (**B–H**) Intralibrary phylogenetic trees for libraries containing ≥5 *parvovirus* sequences, based on NS1 protein sequences. (**I–N**) Comparison of *parvovirus* sequence abundance among different penguin species. Box plots show the median and interquartile range, with individual libraries represented as dots. Statistical significance is indicated by asterisks (*P < 0.05; **P < 0.01).

In this study, 83 nearly complete genome sequences of Parvoviridae were identified. Their NS1 proteins showed amino acid similarities ranging from 39.46% to 64.53%. Phylogenetic analysis classified these sequences into four major clades: Parvovirinae (*n* = 1), Densovirinae (*n* = 1), Hamaparvovirinae (*n* = 79), and unclassified Parvoviridae (*n* = 2) (Fig. 4A). Notably, 79 sequences within the Hamaparvovirinae clade clustered with Chaphamaparvovirus and were most closely related to viruses from Tasmanian devils and various bird species. Their NS1 similarities were all below 64.53%, suggesting that they likely represent novel species within this genus. Two sequences (qFe06-k141-1 and qFe04-k141-6) exhibited less than 30% NS1 similarity to any defined genera, indicating that they may represent a new subfamily or genus (Fig. 4A).

Host distribution analysis indicated that among the 83 parvovirus sequences, 46 originated from *S. humboldti*, 6 from *P. papua*, 29 from *P. adeliae*, and 2 from *A. forsteri*. Seven libraries contained more than five sequences each: five libraries from *S. humboldti* (qFe01–qFe05, containing 8, 6, 13, 6, and 13 sequences, respectively) and two libraries from *P. adeliae* (qFe13 and qFe15, containing 8 and 19 sequences, respectively). Phylogenetic trees constructed for these seven libraries individually (Fig. 4B through H) revealed significant genetic divergence among sequences within each library, ruling out the possibility of duplicates. Concurrently, to compare the relationships between the parvovirus sequences obtained in this study and their closest reference sequences in the NCBI database, an amino acid sequence similarity matrix was generated (Fig. S3). This matrix displays the levels of amino acid similarity among different sequences, showing that the sequences obtained in this study generally exhibited low overall similarity to known reference sequences, suggesting that they may represent novel evolutionary lineages. In the comparison of sequence abundance among different penguin species (Fig. 4I through N), the differences between *S. humboldti* and both *P. papua* and *A. forsteri* were significant, while differences among other groups were not significant. This result indicates that multiple parvovirus co-detections were common in *S. humboldti* individuals. Although a relatively high number of Parvoviridae were detected in some *P. adeliae* libraries, co-detection was not a widespread characteristic in this species overall.

#### Evolution and new species of Anelloviridae

Anelloviridae (AVs) are circular single-stranded DNA viruses with a genome length of approximately 2.1–3.9 kb, typically containing three overlapping open reading frames (ORFs): ORF1 encodes the major capsid protein VP1, while VP2 and VP3 encode auxiliary proteins (25). Among these, the genus Gyrovirus is a significant group within this family. It was initially classified under Circoviridae and reclassified into Anelloviridae in 2017 (26). Previous studies have shown that Gyrovirus can cause disease in avian hosts, with the most representative being chicken anemia virus (CAV), which induces severe anemia and immunosuppression in young chicks and often leads to high mortality in the absence of maternal antibody protection (27). Additionally, Gyrovirus has been associated with respiratory distress syndrome (RDS) in yellow-eyed penguins, causing pulmonary lesions (26). In this study, five novel Gyrovirus genome sequences were identified and characterized from the penguin fecal virome.

**Fig 5:**
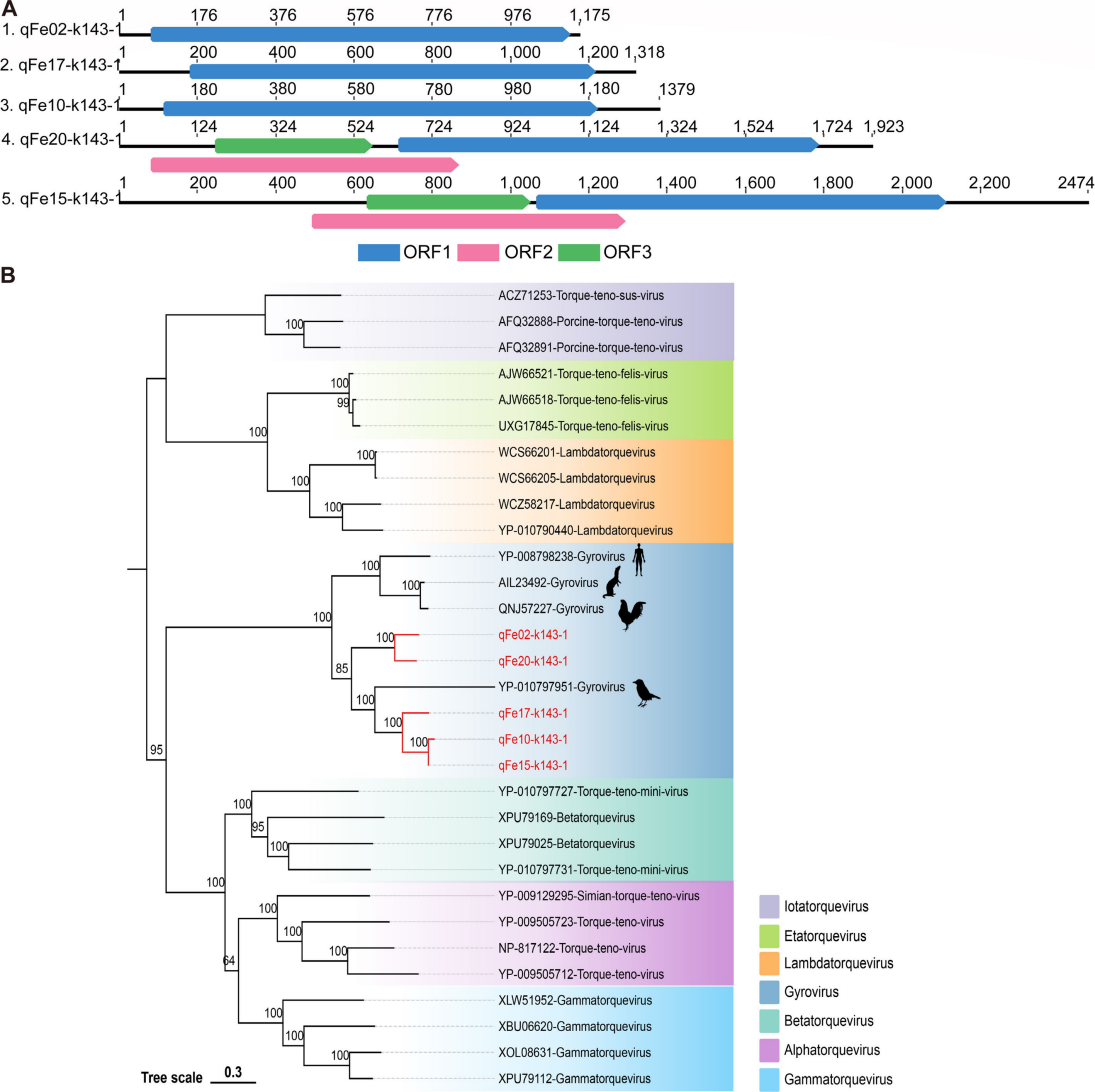
Genomic organization and phylogenetic analysis of anelloviruses detected in penguins. (**A**) Genomic organization of the five anelloviruses identified in this study. Viral open reading frames (ORFs) are shown in different colors, and arrows indicate gene orientation. (**B**) Phylogenetic tree based on the amino acid sequences of ORF1 from the five anelloviruses identified in this study and representative reference strains. The sequences identified in this study are highlighted in red.

A total of five genome sequences belonging to the family Anelloviridae, each containing a complete ORF1, were obtained in this study. They originated from *Spheniscus humboldti* (qFe02-k143-1), *Pygoscelis papua* (qFe10-k143-1), *Pygoscelis adeliae* (qFe15-k143-1), and two sequences from *Aptenodytes forsteri* (qFe17-k143-1 and qFe20-k143-1) (Fig. 5A). Phylogenetic analysis based on the amino acid sequences of the conserved ORF1 region showed that these sequences clustered within the Gyrovirus clade (Fig. 5B) and were most closely related to gyroviruses of human, chicken, ferret, and *Ferruginous-backed Antbird*. Sequence alignment indicated that the ORF1 amino acid similarity among these penguin-origin gyroviruses ranged from 41.76% to 47.52%, which is below the 60% threshold for species demarcation but above the 35% threshold for genus demarcation, suggesting they likely represent novel Gyrovirus species from different penguin hosts.

#### Identification and phylogeny of Circoviridae

Circoviridae is a family of small viruses with a single-stranded circular DNA genome, typically ranging from 1.8 to 2.1 kb in length. The genome contains two major open reading frames (ORFs): one encoding the replication-associated protein (Rep) and the other encoding the capsid protein (Cap) (28). Based on molecular characteristics and phylogenetic analysis, this family is currently divided into two genera: Circovirus and Cyclovirus (29). Circoviridae have been confirmed to infect various birds, fish, and mammals. Representative viruses include *Porcine* Circovirus *type 2* (PCV2) and Beak and Feather Disease Virus (BFDV). PCV2 is associated with Postweaning Multisystemic Wasting Syndrome (PMWS) (30), while BFDV causes Psittacine Beak and Feather Disease (PBFD) (31). Additionally, other viruses, such as *Duck* Circovirus (DuCV) and *Finch* Circovirus (FiCV), are associated with clinical symptoms similar to PBFD, including feather abnormalities (32).

**Fig 6:**
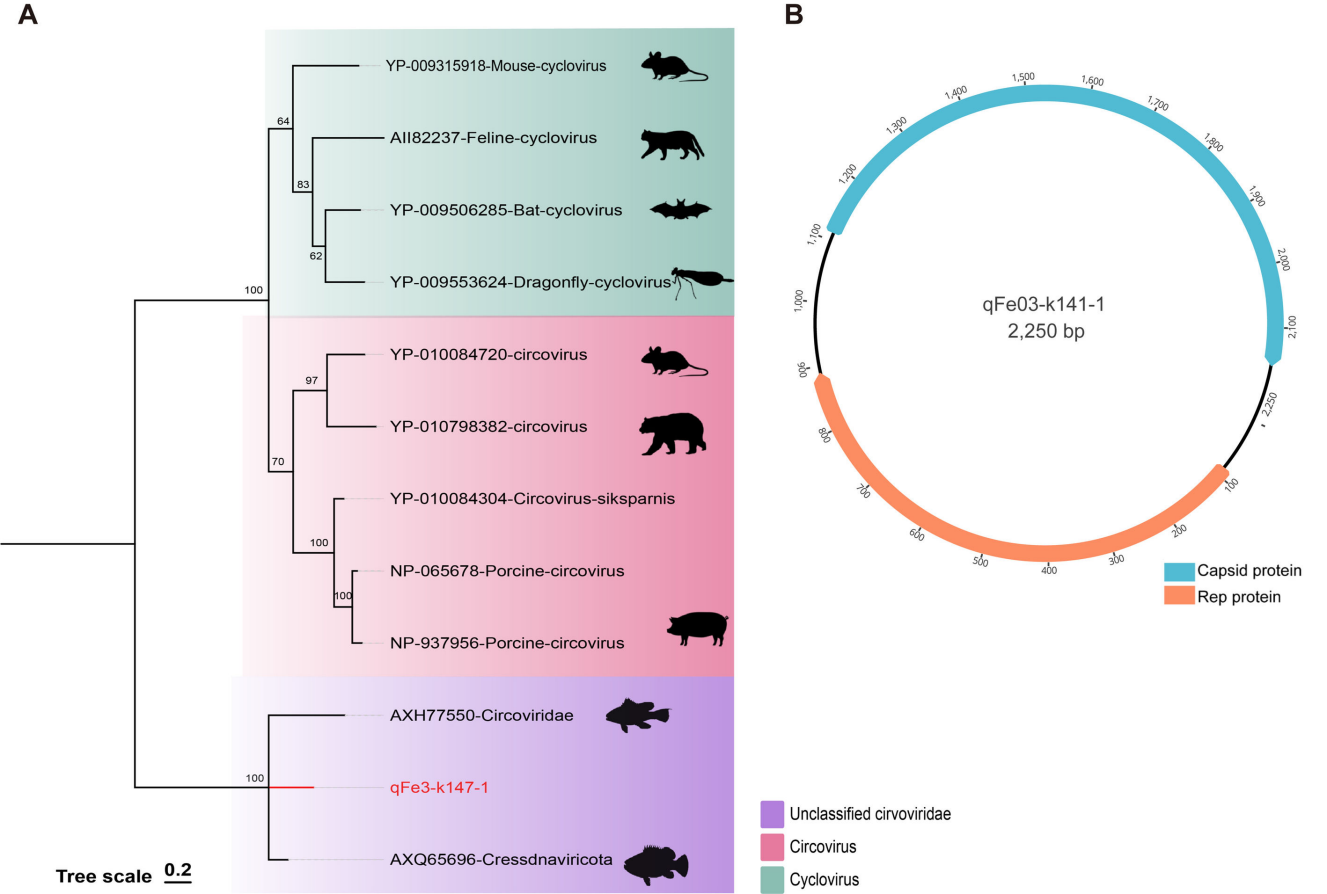
The genomic organization and phylogenetic analysis of circovirus*es* identified in *Spheniscus humboldti*. (**A**) Phylogenetic analysis based on the Rep proteins of the Circovirus identified in this study. (**B**) The genomic organization of the Circovirus identified in *Spheniscus humboldti*. Viral encoding proteins of the Circovirus are marked with different colors.

In this study, a complete genome belonging to the family Circoviridae, designated qFe03-k147-1, was identified from a fecal sample of *Spheniscus humboldti*. The genome has a total length of 2,250 bp (Fig. 6B). Phylogenetic analysis based on the amino acid sequence of the Rep protein revealed that this sequence did not cluster with known genera Circovirus or Cyclovirus. Instead, it formed a distinct branch with an unclassified Circoviridae sequence from red snapper (GenBank no. AXH77550), exhibiting a sequence similarity of 57%, which is substantially below the 75% threshold for species demarcation (Fig. 6A). These results suggest that qFe03-k147-1 represents a novel member of the family Circoviridae. Given the predatory feeding behavior of *S. humboldti*, it is hypothesized that this virus may have originated from infected fish and entered the penguin’s intestinal tract through the dietary route.

#### Phylogeny and genetic similarity of Picornaviridae

Picornaviridae are small, non-enveloped icosahedral viruses with a positive-sense single-stranded RNA genome approximately 6.7–10.1 kb in length (33). According to the ICTV 10th Report, this family is currently divided into 47 genera and 110 confirmed species, exhibiting a broad host range and infecting various vertebrates (34). Avian Picornaviridae are commonly found in orders such as *Anseriformes* (ducks, geese), *Galliformes* (chickens, quails), and *Passeriformes* (robins, thrushes), causing diseases like hemorrhagic hepatitis, avian encephalomyelitis, and catarrhal enteritis (35). Recent research indicates the presence of various virus families in Antarctic animals, suggesting that penguins may play a role in the ecology and transmission of pathogens such as influenza viruses (36, 37).

**Fig 7:**
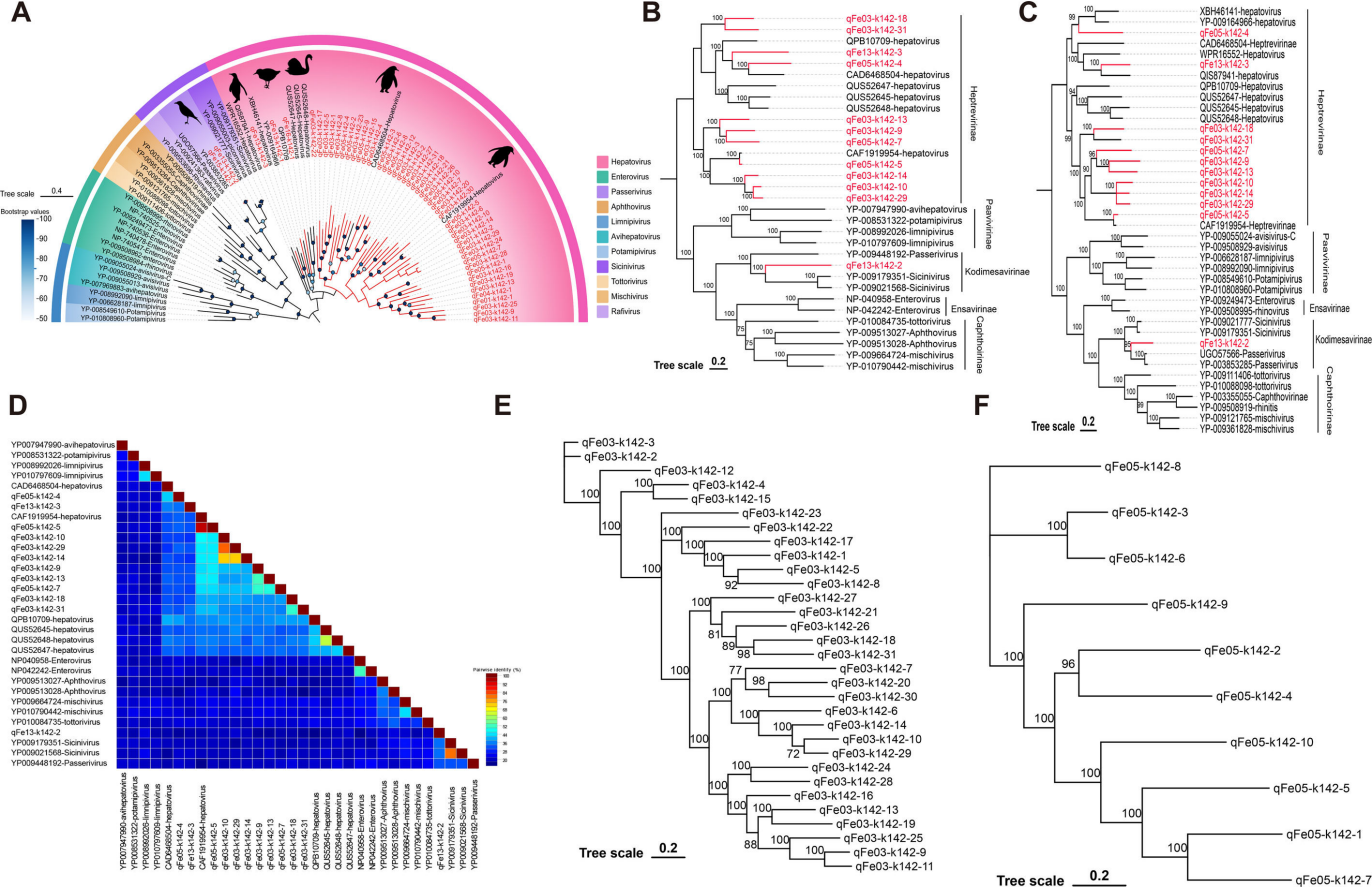
Phylogenies of the Picornaviridae family identified in penguins. (**A**) Bayesian inference tree based on the amino acid sequences of RdRp from viruses in the Picornaviridae family, including representative strains from other genera. (**B–C**) Phylogenetic analysis based on the P1 and 3CD regions of the twelve complete Picornaviridae genomes identified in this study, with reference sequences from the Picornaviridae family. The twelve genomes identified in this study are highlighted in red. (**D**) Pairwise comparison of amino acid sequences of the P1 region from the twelve complete Picornaviridae genomes with representative strains, based on the complete P1 protein sequences. (**E–F**) Phylogenetic trees based on RdRp showing the evolutionary branches of picornaviruses found in the *Humboldt* penguin libraries qFe03 and qFe05.

In this study, a total of 49 Picornaviridae-related sequences were obtained, 12 of which represented complete genomes. Phylogenetic analysis based on the RdRp amino acid sequences revealed that these sequences primarily clustered within two subfamilies, Heptrevirinae and Kodimesavirinae; 47 sequences showed closer affinity to Hepatovirus, while two were more closely related to Passerivirus (Fig. 7A). Comparison with reference sequences indicated that the RdRp amino acid similarity of these newly obtained sequences ranged from 38.62% to 66.55%. Further phylogenetic trees based on the P1 and 2C+3 CD regions, respectively, showed that most sequences maintained consistent topological positions in both trees. However, sequence qFe13-k142-2 clustered closer to Passerivirus in the 2C+3 CD tree but grouped with Sicinivirus in the P1 tree, suggesting a potential recombination event (Fig. 7B and C). Amino acid sequence similarity matrix analysis further supported this finding: except for qFe13-k142-2, which exhibited >35% similarity to a Sicinivirus reference strain, the other 11 complete genomes showed >34% similarity to Hepatovirus (Fig. 7D). Notably, Hepatovirus and Passerivirus are associated with hepatitis A and enteritis, respectively, suggesting that these novel penguin-origin Picornaviridae may have animal and public health significance. In *S. humboldti* sample libraries, we identified 31 and 10 *Picornavirus* sequences in samples qFe03 and qFe05, respectively (Fig. 7E and F), suggesting potential co-detection, although this was not widespread.

#### Phylogeny and prevalence of Caliciviridae

Caliciviridae is a family of non-enveloped, positive-sense single-stranded RNA viruses with a genome length of 6.4–8.5 kb, containing multiple open reading frames (ORFs) that encode structural and non-structural proteins. The viral particles exhibit icosahedral symmetry with a diameter of 27–40 nm (38). This family is divided into 11 genera: Lagovirus, Norovirus, Nebovirus, Recovirus, Sapovirus, Valovirus, and Vesivirus (infecting mammals); Bavovirus and Nacovirus (infecting birds); and Minovirus and Salovirus (infecting fish). Unclassified Caliciviridae viruses have been detected in a wide range of animal hosts, including geese, yellowtail fish, rainbow trout, lampreys, frogs, and various Australian bird species (39).

**Fig 8:**
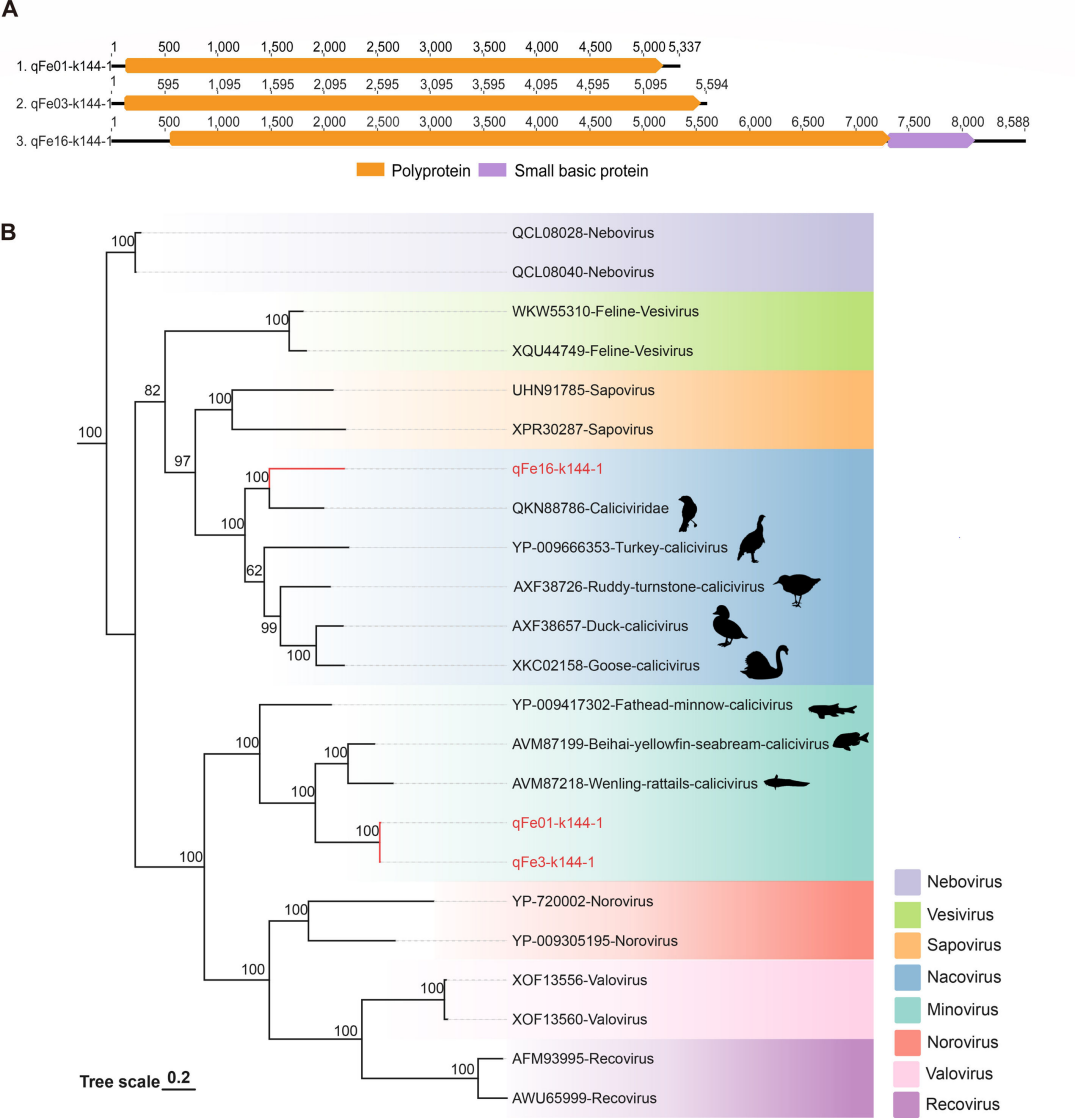
Phylogenies of the Caliciviridae family identified in penguins. (**A**) Genomic organization of the three Caliciviridae genomes identified in this study. Viral encoding proteins are color-coded, with arrows indicating gene coding direction. (**B**) Bayesian inference tree based on the amino acid sequences of the CP protein from the Caliciviridae family. Viruses identified in this study are highlighted with red branches or red labels. Taxonomic clusters are colored differently, and taxon names are shown on the right. The tree scale represents amino acid substitutions per site.

In this study, three nearly complete viral genomes belonging to Caliciviridae were obtained: qFe01-k144-1, qFe03-k144-1, and qFe16-k144-1, with genome lengths of 5,337, 5,594, and 8,588 nucleotides and GC contents of 50.9%, 50.1%, and 50.3%, respectively. Complete polyprotein sequences were assembled for these genomes, encoding 1,686, 1,808, and 2,257 amino acids, respectively (Fig. 8A). Phylogenetic analysis based on the capsid protein (CP) indicated that qFe01-k144-1 and qFe03-k144-1 shared the highest similarity with a Calicivirus from *Acanthopagrus latus*, with amino acid identities of 58.16% and 54.13%, respectively, and clustered within the Minovirus branch. qFe16-k144-1 showed the highest similarity (48.88%) with a Calicivirus from *Eophona migratoria* and clustered within the Nacovirus branch (Fig. 8B).

#### Microviridae diversity and host enrichment

**Fig 9:**
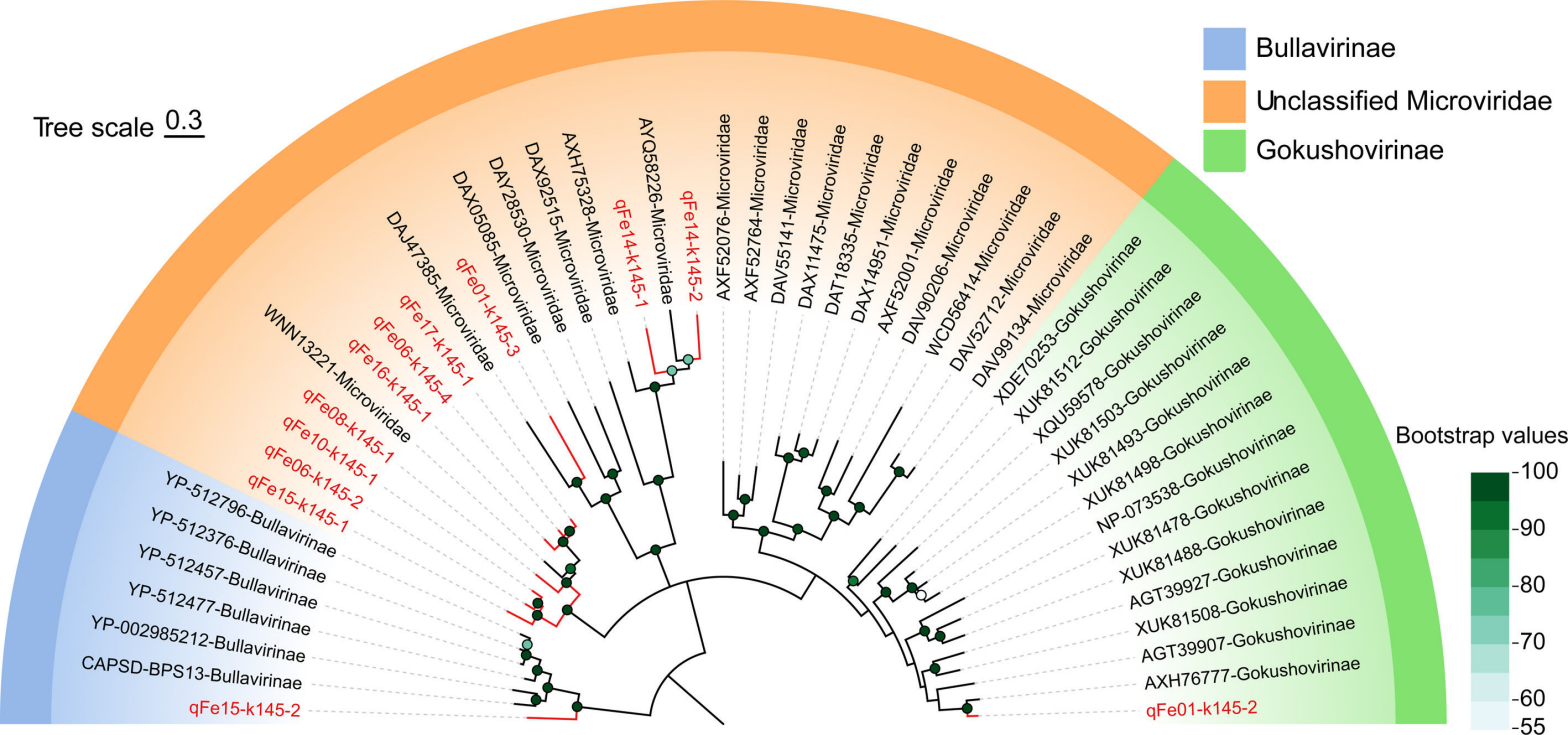
The phylogeny of Microviridae identified in feces of penguins. Bayesian inference tree established based on amino acid sequences of MCP of Microviridae. The 12 Microviridae identified in this study are highlighted using red font.

Microviridae is a family of single-stranded DNA bacteriophages characterized by small circular genomes, approximately 4.4–6.7 kb in length, encoding three to five functional proteins. According to the latest classification by the International Committee on Taxonomy of Viruses (ICTV), this family is divided into three subfamilies: Gokushovirinae, Bullavirinae, and Alpavirinae, along with several unclassified evolutionary clades (40). In this study, 12 sequences containing complete major capsid protein (MCP) regions were identified. BLASTx analysis revealed that all sequences exhibited significant homology with known Microviridae members. Phylogenetic analysis based on MCP amino acid sequences showed that these new sequences formed multiple distinct branches. Among them, qFe15-k145-2 shared 52.33% similarity with *CAPSD-BPS13* (Bullavirinae) from *Escherichia coli*; qFe01-k145-2 showed 84.78% similarity with *AXH76777* (Gokushovirinae) derived from minnow tissue. The remaining 10 sequences clustered within unclassified clades, suggesting they represent novel evolutionary lineages. This distribution indicates the presence of previously unclassified Microviridae evolutionary clades in the penguin gut (Fig. 9).

#### Caudoviricetes phylogeny and diversity

**Fig 10:**
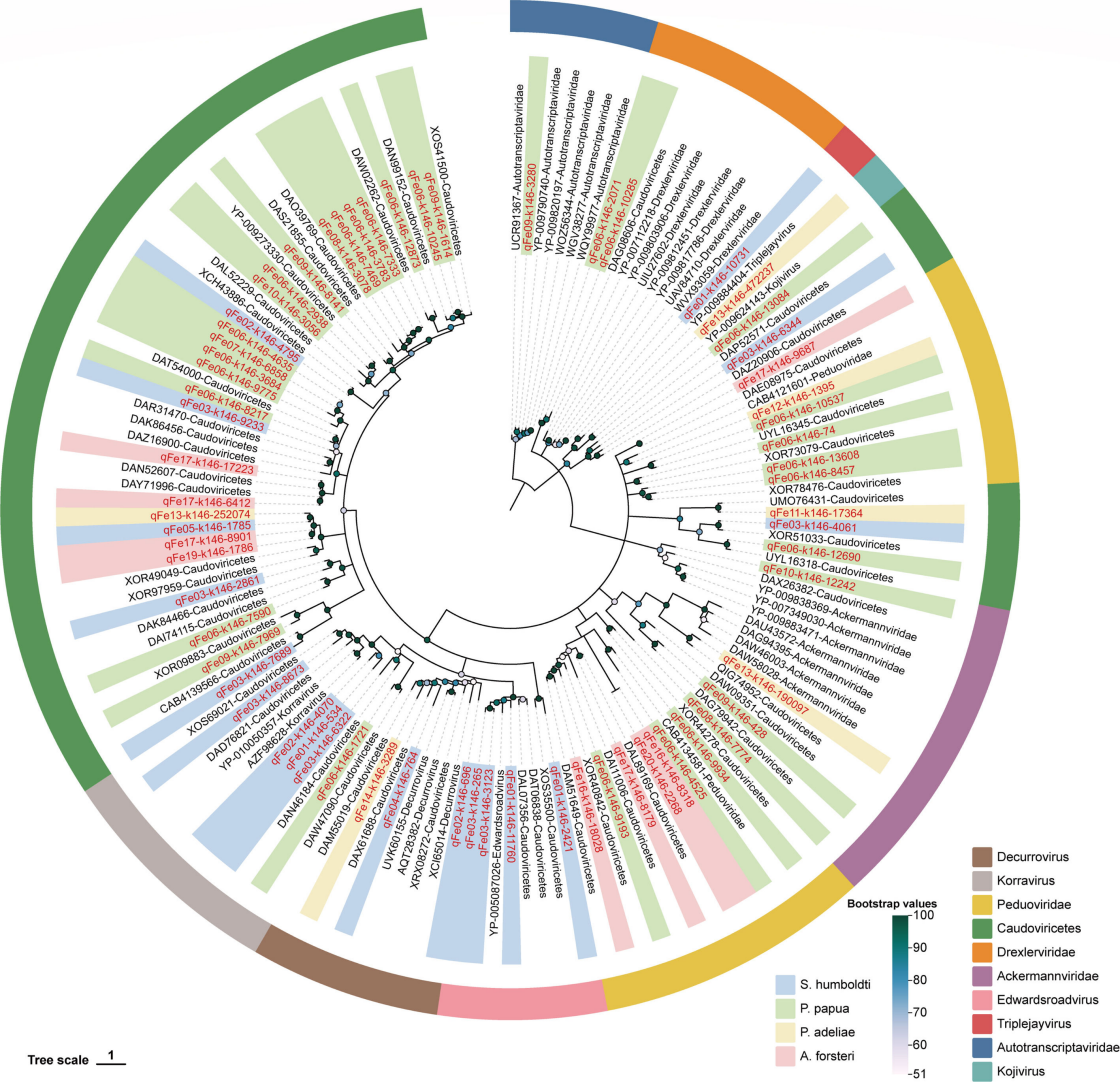
The phylogeny of Caudoviricetes identified in feces of penguins. Bayesian inference tree based on amino acid sequences of TerL of Caudoviricetes. Representative strains from all families in Caudoviricetes are included and marked with the color coding in the key on the bottom right. The viruses identified in this study are represented by sectors filled with four different colors according to penguin species. The scale bar indicates the amino acid substitutions.

The gut virome is predominantly composed of bacteriophages, with Caudoviricetes being the most significant double-stranded DNA phages, playing crucial roles in microbial ecology and host immune regulation (41). To investigate the diversity and evolutionary relationships of Caudoviricetes in the penguin gut, we selected the conserved terminase large subunit (TerL) gene as a molecular marker for phylogenetic analysis. A total of 66 Caudoviricetes sequences were obtained. Phylogenetic analysis revealed that these sequences belonged to multiple families and genera, including Decurrovirus (*n* = 3), Korravirus (*n* = 5), Peduoviridae (*n* = 13), Drexlerviridae (*n* = 2), Ackermannviridae (*n* = 4), Edwardsroadvirus (*n* = 4), Triplejayvirus (*n* = 1), Autotranscriptaviridae (*n* = 1), Kojivirus (*n* = 1), and unclassified Caudoviricetes clades (*n* = 32). The constructed phylogenetic tree illustrated the relationships between newly discovered Caudoviricetes and known families. Most novel sequences did not cluster within known families but were distributed across unclassified Caudoviricetes clades. This evolutionary pattern indicates the presence of a highly diverse community of Caudoviricetes in the penguin gut, with significant genomic variation, suggesting these phages play important roles in maintaining the homeostasis and ecological functions of the penguin gut microbiota (Fig. 10).

## DISCUSSION

Research on the distribution and diversity of viruses in birds has been a significant focus in viral ecology and public health. Previous studies have shown that birds are not only important natural reservoirs for various novel viruses but may also play a critical role in cross-species transmission. Penguins, as a unique group of seabirds, have not been sufficiently studied regarding the composition and diversity of their gut viral communities. In this study, we detected 219 viral sequences that may represent novel viral lineages across 20 libraries from four penguin species (*Spheniscus humboldti*, *Pygoscelis papua*, *Pygoscelis adeliae*, and *Aptenodytes forsteri*). Over 94% of these sequences exhibited less than 80% similarity to known viruses, indicating the presence of substantial “viral dark matter” in the penguin gut virome (20). According to the current ICTV classification framework, viral classification in the families examined in this study—including Parvoviridae, Picornaviridae, Caliciviridae, Anelloviridae, and Circoviridae—is generally evaluated based on the amino acid similarity of key proteins together with phylogenetic relationships. Therefore, the novelty of the viral sequences detected here was assessed primarily through the amino acid similarity of hallmark proteins and phylogenetic analyses rather than relying solely on a single similarity threshold. This virome diversity provides new insights into the evolution and ecological functions of the viral communities in the penguin gut.

Alpha diversity analysis revealed no significant differences in viral diversity at the family level among the four penguin species’ gut viromes, but significant differences were observed at the genus and species levels. At the genus level, a difference was found between *P. papua* and *A. forsteri*; at the species level, *S. humboldti* and *A. forsteri* differed significantly. Principal coordinates analysis (PCoA) further revealed significant differences in beta diversity among penguin populations. Specifically, *P. adeliae* exhibited considerable inter-individual variation in its viral community, while *S. humboldti* showed a more stable structure. These differences may be linked to host factors such as immune system, diet, and habitat. Relative abundance stacked bar plots demonstrated distinct differences in community composition among species: Parvoviridae dominated in *S. humboldti*, Microviridae was enriched in *P. papua*, Caliciviridae was prominent in *A. forsteri*, and the viral community of *P. adeliae* was relatively balanced. UpSet plot analysis showed that the four groups shared only 25 species (accounting for 13%–25% of their respective species), indicating significant host-associated differences and niche differentiation.

Among the detected DNA viruses, Parvoviridae represented the predominant group, accounting for the largest proportion of DNA viral sequences. A total of 83 near-complete Parvoviridae genome sequences were recovered, with NS1 amino acid similarities ranging from 39.46% to 64.53%. These values fall below the 85% threshold for species demarcation, suggesting that they likely represent multiple novel species (42). In *S. humboldti*, Parvoviridae were particularly abundant, exhibiting a pattern of multi-lineage co-existence, which may facilitate genomic recombination and the emergence of new variants, potentially enhancing viral transmissibility and pathogenicity, thus increasing outbreak risks. In addition to Parvoviridae, five Gyrovirus genome sequences from the family Anelloviridae were identified, each containing a complete ORF1 with amino acid similarities ranging from 41.76% to 47.52%, indicating the potential existence of several novel species. These sequences showed phylogenetic relationships with poultry-associated viruses, suggesting possible cross-host transmission. Furthermore, a complete genome from the family Circoviridae (qFe03-k147-1) was identified in a fecal sample of *S. humboldti*. Its Rep protein shared 57% amino acid similarity with an unclassified Circovirus from red snapper, suggesting it is likely an unclassified or novel member of Circoviridae. Considering penguins’ predatory behavior, this virus may originate from dietary exposure.

Among the RNA viruses, members of the family Picornaviridae were particularly prominent. Notably, Hepatovirus strains were detected at high levels in apparently healthy penguins, with occasional co-detection observed in individual *S. humboldti* (e.g., qFe03, qFe05). This indicates that penguins, even without clinical symptoms, may harbor potentially pathogenic viruses. This “subclinical carriage” suggests that certain Hepatovirus strains could retain pathogenic potential despite the absence of overt disease. Similar phenomena have been reported in humans and poultry, where Hepatovirus strains persist in healthy hosts and proliferate under immunosuppression or environmental stress (43). Therefore, hepatoviruses in penguins should be considered potential risk factors rather than harmless inhabitants. Future studies should integrate metatranscriptomic sequencing, quantitative PCR, and histopathological examinations to confirm replication activity and tissue tropism of these viruses. Additionally, while Caliciviridae are widely distributed among fish, avian, and mammalian hosts, their presence in penguins has been underexplored. In this study, we characterized three nearly complete Caliciviridae-related genomes from penguin cloacal samples. Among these, qFe01-k144-1 and qFe03-k144-1 showed high amino acid similarity to piscine-origin caliciviruses, suggesting possible dietary or environmental origins of these viral sequences, while qFe16-k144-1 clustered with avian-origin caliciviruses, highlighting phylogenetic relatedness to other avian-associated caliciviruses.

This study also revealed the widespread presence of a diverse bacteriophage community in the penguin gut. Peduoviridae was evenly distributed across the four penguin species, potentially representing a core component of the gut virome, while Microviridae was particularly enriched in *P. papua*. Phylogenetic analysis based on the terminase large subunit (TerL) identified 66 Caudoviricetes sequences, spanning multiple families and genera, with a substantial number clustering within unclassified clades. This indicates a highly diverse phage community in the penguin gut. Bacteriophages play crucial roles in regulating bacterial communities and may indirectly influence the colonization and replication of eukaryotic viruses. Through lytic/lysogenic cycles, phages modulate bacterial abundance and metabolic output, altering the gut environment and potentially facilitating or inhibiting eukaryotic virus infections. Therefore, changes in the phage community could serve as an early indicator of penguin gut health.

Despite providing valuable insights into the diversity and host-associated differences of the penguin gut viral community, this study has certain limitations. First, the sample size was relatively small, including only four penguin species from a single zoo, which may not fully represent viral diversity across different habitats and ecological conditions. However, the sampling site was selected because multiple penguin species are maintained at this facility under relatively consistent husbandry and dietary conditions, allowing controlled cross-species comparisons. Future studies should expand the sample size to include more species and conduct virome monitoring across various habitats and seasons. Second, viral metagenomic approaches detect viral nucleic acids but cannot determine whether the detected viruses are actively replicating in the host or merely passing through the digestive tract. Consequently, the ecological roles and pathogenic potential of these viruses remain unclear. Future investigations incorporating virus isolation, longitudinal sampling, host immune response analyses, and studies of wild penguin populations will be essential to better understand the biological significance and transmission dynamics of penguin-associated viruses.

## MATERIALS AND METHODS

### Sample collection and processing

In November 2024, we collected a total of 20 cloacal samples from five *Spheniscus humboldti*, five *Pygoscelis papua*, five *Pygoscelis adeliae*, and five *Aptenodytes forsteri* at the Chimelong Ocean Kingdom. Under the guidance of zoo staff, we observed the penguins from a distance to minimize disturbance to their normal activities. Sterile disposable gloves and swabs were used during sample collection. Samples were collected immediately after defecation, and the sampling sites were cleaned thoroughly to minimize any impact on the habitat. Collected samples were transferred immediately into pre-sterilized cryotubes, transported on dry ice, and stored at −80°C. For processing, samples were thawed, resuspended in 1 mL of Dulbecco’s phosphate-buffered saline (DPBS), and then shaken on an orbital shaker for 5 minutes, followed by incubation at 4°C for 30 min, with shaking repeated three times. The samples were then centrifuged at 12,000 × *g* for 5 min at low temperature to remove large non-viral particles and other impurities. The supernatant was collected into 1.5 mL microcentrifuge tubes and stored at −80°C for further use.

### Sample preparation and library construction

A 500 µL aliquot of supernatant from each fecal sample was filtered through a 0.45 µm filter (Millipore, Darmstadt, Germany) to remove eukaryotic and bacterial cell-sized particles, yielding at least 200 µL of filtrate enriched with viral particles (44). This pore size was selected as a commonly used approach in viral metagenomics to reduce cellular and bacterial contamination while allowing most viral particles to pass through. Although very small viral particles may potentially be lost during filtration, this step helps improve viral nucleic acid enrichment and the overall quality of downstream sequencing libraries. The filtrate was then treated with DNase and RNase enzymes (Turbo DNase, Thermo Fisher Scientific, MA, USA; BaselineZERO DNase, Epicenter, WI, USA; RNase A, Thermo Fisher Scientific) and incubated at 37°C for 60 min to digest non-viral nucleic acids (45). Halfway through the enzymatic digestion, the tubes were inverted to mix, ensuring complete nucleic acid digestion. Following the enzymatic treatment, the remaining nucleic acids were extracted using the QIAamp Viral RNA Mini Kit (QIAGEN) according to the manufacturer’s instructions. A reverse transcription reaction was performed to convert viral RNA into DNA. A two-step RT-PCR was conducted using the SuperScript III Reverse Transcriptase Kit, followed by Klenow Fragment polymerase (New England Biolabs) to generate double-stranded DNA (dsDNA). The dsDNA products were used for library construction. Twenty libraries were prepared using the Nextera XT DNA Sample Preparation Kit (Illumina), and their quality was assessed by agarose gel electrophoresis and an Agilent 2100 Bioanalyzer. PCR amplification cycles were limited to 16 to reduce duplication bias and preserve library diversity. Based on the band distribution and intensity, an appropriate amount of product was extracted for purification, ensuring compatibility with the Illumina NovaSeq 6000 sequencing platform. Sequencing was performed using a 150 bp paired-end read length mode with dual indexing for each sample pool.

### Quality control

Strict standardized precautions were followed to ensure high-quality library preparation and reliable experimental results, effectively avoiding cross-contamination and nucleic acid degradation (46). Aerosol-filter pipette tips were used throughout the experiment to minimize the risk of cross-contamination. All laboratory consumables, including microcentrifuge tubes and pipette tips, were screened and certified to be nuclease-free (RNase and DNase) to prevent enzymatic degradation of nucleic acids. To further preserve nucleic acid integrity, all samples were dissolved in diethyl pyrocarbonate (DEPC)-treated water to inactivate potential nuclease activity. Additionally, an RNase inhibitor was added during sample processing, ensuring the high quality of nucleic acid samples and experimental accuracy.

### Viral metagenomic analysis

Following sequencing, the 150 bp paired-end reads generated by the NovaSeq platform were demultiplexed according to sample-specific dual-index barcodes using Illumina bcl2fastq software (v2.20, default parameters). An in-house analysis pipeline running on a 32-node Linux cluster was used for data processing. Reads were aligned to the host genome using Bowtie2 (v2.4.5, --very-sensitive-local mode) to remove potential host-derived sequences (47). Reads with ≥90% nucleotide identity and ≥90% coverage to the host genome were discarded. Duplicates were identified as reads with identical bases from positions 5 to 55, with only one randomly selected copy retained. Adapter trimming and quality filtering were performed using Trim Galore (v0.6.5) with parameters --phred33, --length 100, --stringency 3, --paired (48). After quality control, *de novo* assembly was performed using MEGAHIT (v1.2.9, parameters: -min-contig-len 200 -t 24) (49). The resulting contigs and unassembled reads were compared against a custom viral protein database using DIAMOND BLASTx (v2.1.8, --evalue 1e-5 --max-target-seqs 10) (50). This database was constructed by integrating the NCBI viral reference proteome (ftp://ftp.ncbi.nih.gov/refseq/release/viral/) and viral protein sequences from the NCBI nr database annotated under viral taxonomy (51).

### Viral community analysis

BLASTx results were imported into MEGAN (MEtaGenome Analyzer, v7.1.1) for taxonomic binning using the Lowest Common Ancestor (LCA) algorithm with default parameters (min-score: 50, top-percent: 10, min-support: 5). Species rarefaction curves were generated in MEGAN v6.22.2 to assess sampling completeness. Community structure was visualized using Bray-Curtis ecological distance matrix, UPGMA, and Principal Coordinates Analysis (PCoA) at the species level. PERMANOVA analysis was performed using the vegan package (v2.6-8) in R v4.3.1 to compare viral community differences among groups. The viral community structure and richness at the family and species levels were visualized using heatmaps, chord diagrams, UpSet plots, and bar charts, generated with the R packages pheatmap (v1.0.12), UpSetR (v1.4.0), and ggplot2 (v3.5.1).

### Viral sequence extension and annotation

Viral contigs potentially originating from the same genome but lacking overlapping regions were merged using Geneious Prime 2024.0.5 software with the “Low Sensitivity/Fastest” parameters. The merged contig was used as the reference sequence and mapped back to the reads under its original barcode. Putative viral open reading frames (ORFs) were predicted using the built-in parameters in Geneious Prime 2024.0.5 (minimum size: 300; genetic code: standard; start codon: ATG) and validated by comparison with related viruses using NCBI BLASTx. Annotations of these ORFs were based on comparisons to the Conserved Domain Database using RPS-BLAST with an E-value cutoff of <10^−5^. Contigs containing hallmark genes of major viral groups were selected for subsequent phylogenetic analysis. The viral hallmark genes used included: the terminase large subunit (TerL) for Caudoviricetes, the major capsid protein (MCP) for Microviridae, non-structural protein 1 (NS1) for Parvoviridae, major capsid protein (ORF1) for Anelloviridae, capsid protein (CP) for Caliciviridae, replication-associated protein (Rep) for Circoviridae, and RNA-dependent RNA polymerase (RdRp) for Picornaviridae.

### Phylogenetic analysis

Phylogenetic analysis was performed using predicted protein sequences of viral hallmark genes, combined with protein sequences of reference strains from different viral taxa downloaded from the NCBI GenBank database based on BLASTx results. Multiple sequence alignment was conducted using MUSCLE v5.1 (BLOSUM62 matrix, gap penalties: −11/−1) implemented in MEGA v11.0.13 (52). TrimAl v1.4.1 (parameter: −automated1) was used for trimming, followed by manual trimming of hypervariable regions. Phylogenetic trees were constructed using Bayesian inference analysis in MrBayes v3.2.7, with the substitution model set to “prset aamodelpr = mixed,” allowing the program to select the best amino acid model(53). The Markov chain was run for 1 million generations, with every 50 generations sampled, and the first 25% of Markov chain Monte Carlo (MCMC) samples discarded as burn-in. MCMC convergence was confirmed via Tracer v1.7.2. The phylogenetic trees were visualized using iTOL (https://itol.embl.de/) (54), Chiplot (https://www.chiplot.online/), and Adobe Illustrator 2021.

## Data Availability

All sequencing data generated in this study have been deposited in the National Genomics Data Center (NGDC; https://ngdc.cncb.ac.cn) under BioProject accession number PRJCA046071. Detailed accession numbers are provided in Tables S1 and S2. All data are publicly accessible without restrictions.

## SUPPLEMENTAL MATERIAL

Figure S1 msphere.00848-25-s0001.pdf Species rarefaction curves of viral communities in penguin samples.

Figure S2 msphere.00848-25-s0002.pdf Species accumulation curves of viral communities in penguin samples.

Figure S3 msphere.00848-25-s0003.pdf Pairwise amino acid similarity matrix of Parvoviridae NS1 proteins.

Table S1 msphere.00848-25-s0004.xlsx Library information for sequencing samples.

Table S2 msphere.00848-25-s0005.xlsx Viral sequences identified in this study.

Table S3 msphere.00848-25-s0006.xlsx Read number and GC content for each sample.

Table S4 msphere.00848-25-s0007.xlsx Number of viral species per sample.

ASM does not own the copyrights to Supplemental Material that may be linked to, or accessed through, an article. The authors have granted ASM a non-exclusive, world-wide license to publish the Supplemental Material files. Please contact the corresponding author directly for reuse.
