# Supplementary material for: Comparative analysis of gut viromes in four penguin species reveals diverse novel viruses and host-associated differences
Source: mSphere. 2026 Jun 30;11(7):e00848-25. doi: 10.1128/msphere.00848-25 (PMC13410756; doi:10.1128/msphere.00848-25)
Supplement: Figure S1 — Species rarefaction curves of viral communities in penguin samples. [file msphere.00848-25-s0001.pdf]

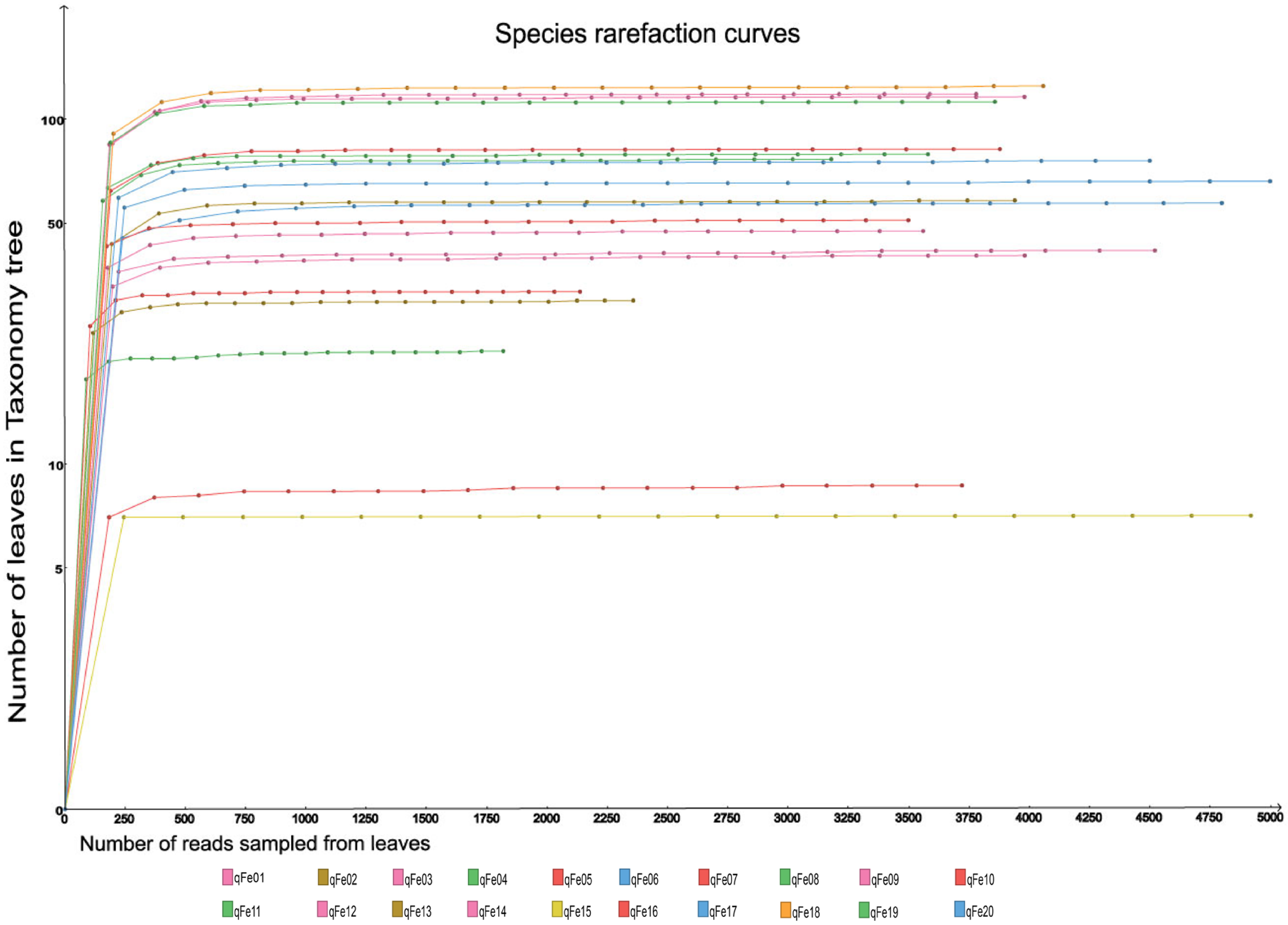

Figure S1. Species rarefaction curves of viral communities in penguin samples. Curves were generated using MEGAN v7.1.1 after log-scale transformation of sequencing reads. Different colors represent individual samples.
