# Supplementary material for: Comparative analysis of gut viromes in four penguin species reveals diverse novel viruses and host-associated differences
Source: mSphere. 2026 Jun 30;11(7):e00848-25. doi: 10.1128/msphere.00848-25 (PMC13410756; doi:10.1128/msphere.00848-25)
Supplement: Figure S2 — Species accumulation curves of viral communities in penguin samples. [file msphere.00848-25-s0002.pdf]

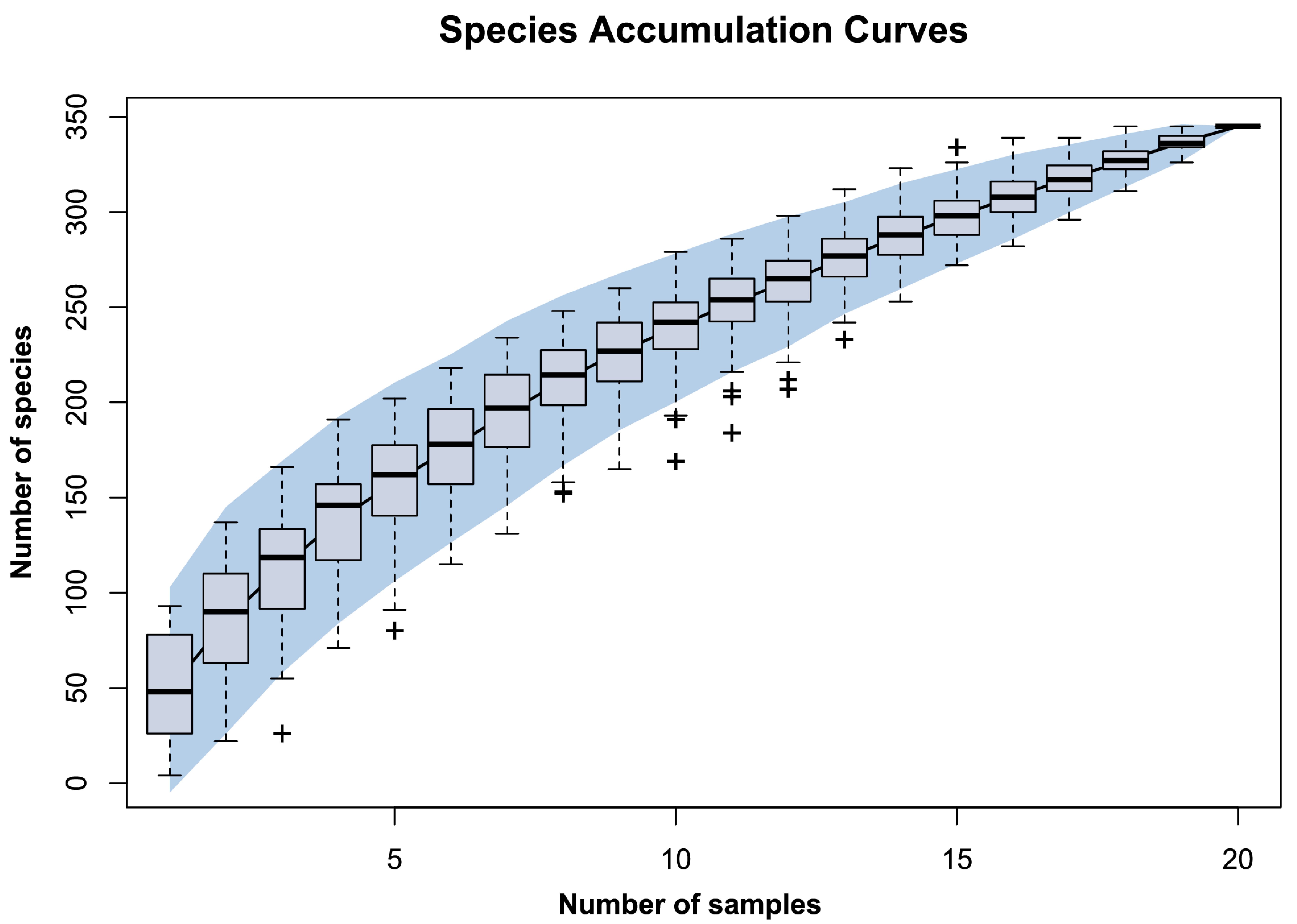

Figure S2. Species accumulation curves of viral communities in penguin metagenomes. Error bars indicate the range of observed values, and the shaded blue area represents the 95% confidence interval.
