# Supplementary material for: Comparative analysis of gut viromes in four penguin species reveals diverse novel viruses and host-associated differences
Source: mSphere. 2026 Jun 30;11(7):e00848-25. doi: 10.1128/msphere.00848-25 (PMC13410756; doi:10.1128/msphere.00848-25)
Supplement: Figure S3 — Pairwise amino acid similarity matrix of Parvoviridae NS1 proteins. [file msphere.00848-25-s0003.pdf]

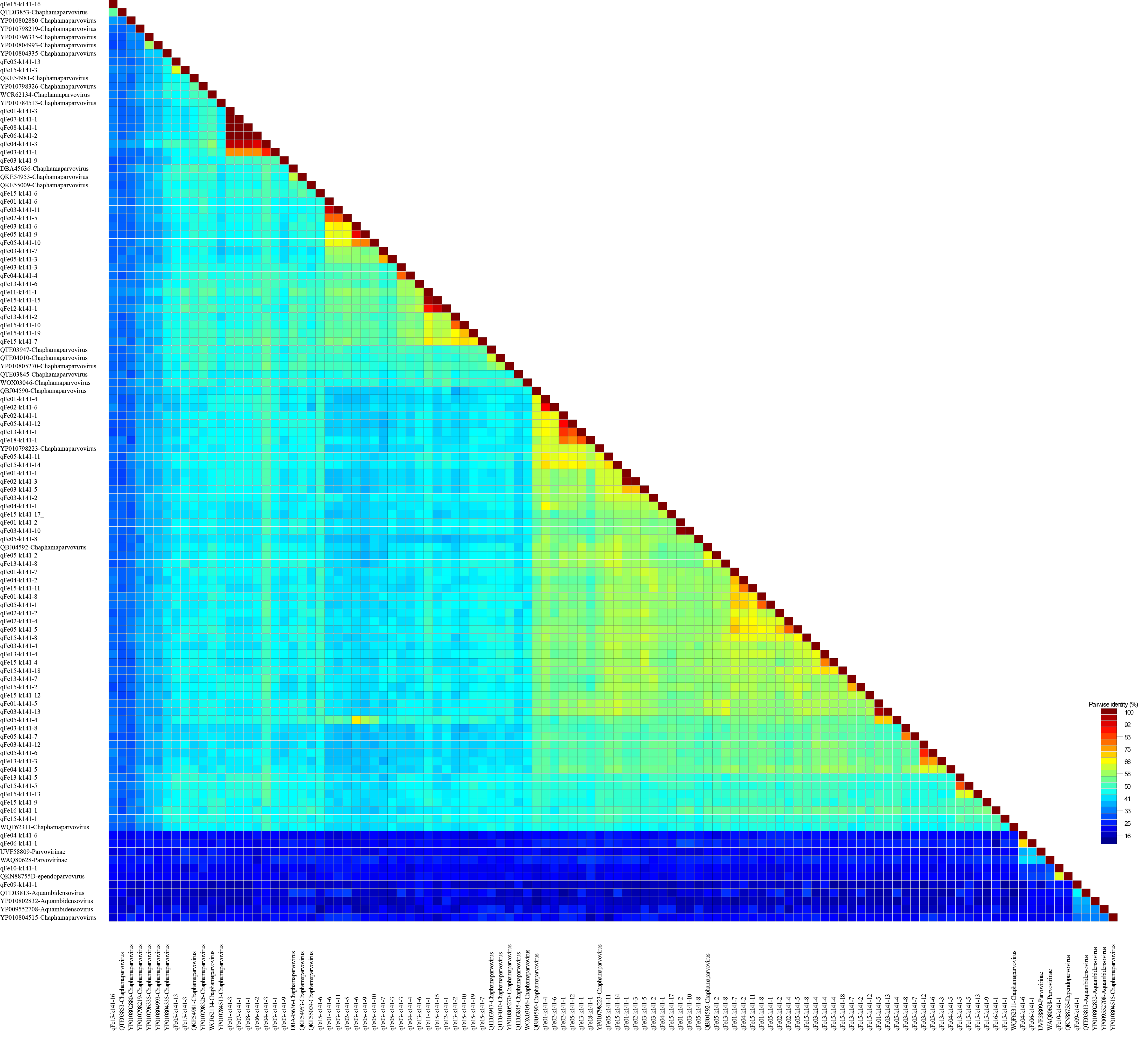

Figure S3. Pairwise amino acid similarity matrix of Parvoviridae NS1 proteins. Pairwise alignments were performed among 83 NS1 sequences identified in this study and representative reference sequences from different subfamilies. Color gradients indicate the percentage of amino acid identity. The identities among newly identified sequences ranged from 39.46% to 64.53%, reflecting their evolutionary relationships with known Parvoviridae.
